# Effect of Dynamic Neuromuscular Stabilization Along With Aquatic Exercises on Pain, Functional Mobility, and Muscle Performance in Bilateral Medial Compartment Knee Osteoarthritis

**DOI:** 10.7759/cureus.109426

**Published:** 2026-05-22

**Authors:** Omkar A Somade, Sandeep Shinde, Mebin S Thomas, Akshaya V Joshi

**Affiliations:** 1 Department of Musculoskeletal Sciences, Krishna College of Physiotherapy, Krishna Vishwa Vidyapeeth (Deemed to be University), Karad, IND

**Keywords:** joint loading, manual muscle testing, multimodal rehabilitation, neuromuscular control, visual analog scale

## Abstract

Background: Bilateral medial compartment knee osteoarthritis (KOA) is characterized by the gradual breakdown of joint structures, which frequently leads to chronic pain, reduced flexibility, and compromised physical function. Although exercise serves as the foundation of non-pharmacological care, there is still a lack of consensus regarding the ideal integration of different therapeutic interventions. Dynamic Neuromuscular Stabilization (DNS) and aquatic therapy have independently demonstrated benefits in improving neuromuscular control and reducing joint loading.

Objective: The objective of this research is to analyze the extent to which integrating aquatic exercises with DNS alters pain perception and functional mobility outcomes for patients suffering from bilateral medial compartment KOA.

Methods: A total of 60 participants with clinically diagnosed bilateral medial compartment KOA were evaluated in a comparative study featuring a pre-test and post-test design. The sample was segmented into two treatment groups: Group A was administered standard physiotherapy, while Group B received a combined regimen of aquatic therapy and DNS. Following an eight-week intervention period of three sessions per week, treatment outcomes were quantified via the Visual Analog Scale (VAS), Timed Up and Go (TUG) test, and Manual Muscle Testing (MMT) of the quadriceps, hamstrings, and hip abductors. Statistical analysis included paired and independent t-tests, with significance set at p<0.05.

Results: Both groups registered statistically significant improvements across all evaluations (p<0.01). However, the integrated approach in Group B resulted in mean improvements that not only demonstrated greater statistical significance but also exceeded the minimal clinically important difference (MCID) thresholds for the VAS and TUG test. Group B exhibited significantly larger decreases in VAS pain scores (p<0.001), greater functional mobility gains in TUG times (p<0.01), and more pronounced MMT grade improvements (p<0.001) compared to Group A, suggesting the potential clinical relevance of the combined intervention at the group level.

Conclusion: A combined regimen of DNS and aquatic therapy may offer enhanced benefits in alleviating pain and enhancing physical function in bilateral medial compartment KOA patients than standard physiotherapeutic interventions. These preliminary findings suggest that integrating specialized neuromuscular stabilization with aquatic training is a promising area for further large-scale investigation.

## Introduction

Bilateral medial compartment knee osteoarthritis (KOA) is a complex, multifactorial, and ubiquitous degenerative joint disease and constitutes a major public health issue on a global scale. Long conceptualized merely as a "wear and tear" condition of the articular cartilage, KOA is now universally recognized as a disease of the entire joint organ. The pathophysiology involves irreversible degradation of the hyaline cartilage, subchondral bone remodeling and sclerosis, osteophyte formation, meniscal degeneration, and persistent, low-grade synovial inflammation [1,2]. Epidemiological data underscore the escalating burden of this condition, currently affecting hundreds of millions globally. This escalating prevalence is propelled by a convergence of demographic and metabolic shifts, predominantly the rapidly aging global population, the rising epidemic of obesity, and increasingly sedentary lifestyles, which collectively place unprecedented mechanical and metabolic stress on weight-bearing joints. Specifically, the medial compartment of the tibiofemoral joint is highly susceptible to this degeneration, as it bears approximately 60-80% of the mechanical load during normal terrestrial gait [3,4].

The clinical presentation of KOA is characterized by a constellation of debilitating symptoms, including chronic, insidious joint pain, localized tenderness, morning stiffness, crepitus, and a progressive reduction in the physiological range of motion. However, pain remains the cardinal symptom that drives individuals to seek medical intervention. In chronic KOA, pain mechanisms are highly complex, originating from peripheral nociceptive sensitization due to localized structural damage and pro-inflammatory cytokine activity (such as IL-1β and TNF-α) within the synovium and often progressing to central nervous system sensitization [2,5]. This persistent pain acts as a primary catalyst for a vicious cycle of physical deconditioning and functional decline. Patients frequently experience severe impairments in functional mobility, making routine activities of daily living, such as ambulating, stair climbing, and sit-to-stand transitions, exceedingly difficult and exhausting [6].

Furthermore, this functional decline is intrinsically linked to profound neuromuscular alterations. A leading contributor to clinical features of KOA is arthrogenic muscle inhibition (AMI), an ongoing reflex inhibition of the periarticular musculature, most notably the quadriceps femoris. AMI prevents full voluntary activation of the muscle, leading to rapid atrophy and severe weakness despite intact neural pathways [7]. Concurrently, structural damage within the joint capsule and ligaments leads to a significant degradation of local mechanoreceptors. This blunts proprioceptive acuity and kinesthetic awareness, depriving the central nervous system of vital feedback required for dynamic joint stability. The culmination of AMI and proprioceptive deficits results in altered gait biomechanics, aberrant joint loading, and a heightened risk of falls, further accelerating the degenerative process [6,8].

While exercise-based rehabilitation is universally championed by international clinical guidelines as the gold-standard, first-line non-pharmacological intervention, traditional land-based conventional physiotherapy presents inherent limitations for certain patient demographics. Closed-kinetic chain, weight-bearing exercises on land can inadvertently increase mechanical compressive forces across the tibiofemoral joint [9]. For patients experiencing acute inflammatory flare-ups, those with advanced radiographic joint degeneration, or individuals with a high body mass index (BMI), this increased loading can exacerbate nociceptive stimulation, increase joint effusion, and reduce exercise compliance, thereby limiting the overall efficacy of the rehabilitation program [4,9].

Consequently, hydrokinesiotherapy or aquatic therapy has gained substantial traction as an optimal therapeutic alternative. Aquatic therapy ingeniously circumvents the mechanical limitations of land-based exercise by leveraging the unique physical and thermodynamic properties of water. According to Archimedes' principle, buoyancy counteracts gravitational forces, creating a microgravity environment that significantly decreases compressive weight-bearing forces. It has been demonstrated that submerging a patient to the level of the anterior superior iliac spine yields an approximate 50% reduction in weight-bearing forces, providing immediate mechanical unloading of the medial compartment of the knee joint [10]. This allows patients to perform pain-free functional movements and gait training that would be intolerable on land. Additionally, hydrostatic pressure exerts a uniform, multidirectional force on the submerged body. This pressure naturally increases with depth and promotes venous and lymphatic return, effectively reducing peripheral edema and joint effusion. Furthermore, the viscosity of water provides accommodating, multiplanar resistance, facilitating safe, low-velocity muscle strengthening without the risk of sudden, jarring impact loads [10,11].

However, while the aquatic environment excellently manages the peripheral biomechanical constraints of KOA, comprehensive rehabilitation must also correct the central, maladaptive neuromuscular patterns that develop as compensatory strategies to chronic pain and AMI. Dynamic Neuromuscular Stabilization (DNS) precisely addresses this critical gap. Developed by the Prague School of Rehabilitation, DNS is an advanced functional rehabilitation paradigm rooted in the scientifically robust principles of developmental kinesiology. This approach is based on the concept that the central nervous system establishes optimal motor control patterns during the first year of infant development [12,13]. DNS focuses on restoring these innate, physiological motor patterns by activating the body's deep intrinsic stabilizers, specifically the local stabilization system, an anatomical complex integrating the thoracic diaphragm, pelvic musculature, transversus abdominis, and deep multifidi [13].

By restoring proper intra-abdominal pressure (IAP) and establishing a stable physiological "punctum fixum" (fixed point) for muscle attachment, DNS facilitates efficient, highly coordinated core-to-limb kinetic chain movements. In the context of bilateral medial compartment KOA, DNS serves to rewire the dysfunctional motor control software, improving joint centration: the optimal alignment of joint surfaces that allows for maximum load transfer with minimal tissue stress. This central approach reduces aberrant compensatory movements, mitigates the effects of AMI, and enhances functional dynamic stability [12,14].

Despite the robust, independent evidence supporting aquatic therapy for biomechanical unloading and DNS for central neuromuscular retraining, there remains a conspicuous paucity of research evaluating their combined, synergistic therapeutic effects. Given their highly complementary mechanisms, that is, aquatic therapy providing the permissive, low-stress environment and DNS providing the optimal neuromuscular "software" for movement, integrating these two distinct modalities presents a compelling theoretical advantage for comprehensive rehabilitation. This study was designed to compare the efficacy of an integrated protocol of DNS and aquatic therapy against conventional physiotherapy in patients with a clinical diagnosis of bilateral medial compartment KOA. The primary objective was to evaluate the combined effectiveness of these interventions on pain reduction. The secondary objective was to assess the impact on functional mobility and muscle performance over an eight-week period. We hypothesized that the integrated DNS and aquatic protocol would result in significantly greater clinical improvements in pain and functional mobility compared to conventional physiotherapy over the eight-week intervention period.

## Materials and methods

Study design

A comparative study was conducted at Krishna Vishwa Vidyapeeth (Deemed to Be University) in Karad, India. The required sample size (n) was determined using the standard equation $\mathrm{n}=\mathrm{Z}^{2}\mathrm{pq}/\mathrm{L}^{2}$. In this calculation, the Z value was established at 1.96 to achieve a 95% confidence interval, the estimated prevalence (p) of KOA was taken as 27.1% [15], q was calculated as 100-p, resulting in 72.9%, and the maximum allowable margin of error (L) was defined as 10%. Consequently, the expected sample size estimated using the formula was 75 participants.

To assess the synergistic impact of DNS and aquatic therapy on pain reduction and functional recovery in patients with bilateral medial compartment KOA, an eight-week comparative study was executed in a clinical physiotherapy setting utilizing a pre-test/post-test framework. The research was carried out from February 1, 2025, to December 31, 2025.

Sample selection

The study enrolled individuals between 40 and 65 years of age presenting with a clinical and radiological diagnosis of bilateral medial compartment KOA, classified as Kellgren-Lawrence grade I or II [16]. Eligibility required a minimum four-week history of chronic knee discomfort during activities such as walking and sit-to-stand transitions. Participants were excluded from the final analysis if they presented with a history of knee replacement surgery, ligament injuries, neurological disorders, cardiovascular instability, or contraindications to aquatic therapy.

A total of 75 individuals were initially evaluated for eligibility to meet the expected sample size requirement. Of these, 15 individuals were excluded from the final study: 10 participants did not meet the specific inclusion parameters, and five participants declined to provide consent. This resulted in a final sample size of 60 participants. Notably, there were no dropouts reported during the eight-week intervention period following the initial allocation. To minimize confounding variables, participants were required to maintain their baseline medication levels. 

**Figure 1:**
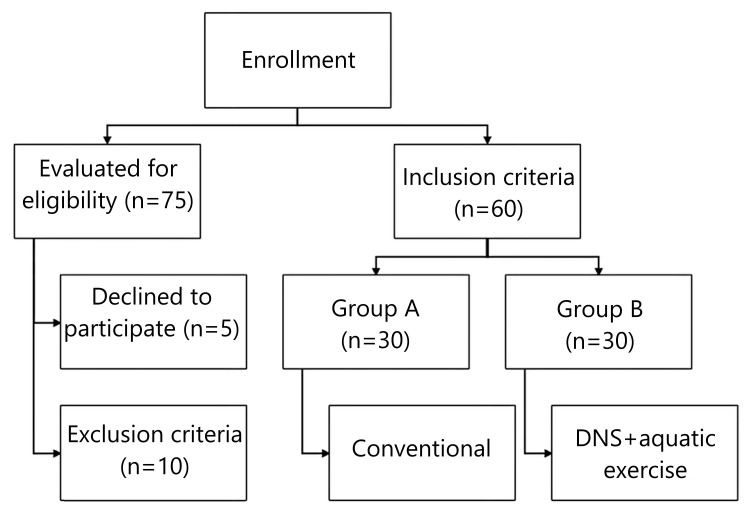
Sampling flowchart and sample size DNS: Dynamic Neuromuscular Stabilization

Due to the inherent nature of the interventions (aquatic vs. land-based), participants and assessors were not blinded to group allocation. To mitigate bias, we implemented procedural blinding: participants were kept unaware of the specific study hypotheses and the alternative group's protocol. Furthermore, strict scheduling prevented inter-group contact to avoid treatment contamination. Participants were randomly assigned to either Group A or Group B using a simple randomization method. To ensure rigorous allocation concealment, group assignments were contained within sequentially numbered, opaque, sealed envelopes (SNOSE). These envelopes were prepared by an independent researcher not involved in participant recruitment or clinical assessment. Each envelope was opened only after a participant's eligibility was confirmed and baseline assessments were completed, ensuring that the treatment allocation remained entirely concealed from the investigator until the point of formal enrollment. This process resulted in two parallel groups of 30 participants each, consisting of Group A receiving conventional physiotherapy and Group B receiving DNS and aquatic therapy (Figure 1).

Ethical considerations

Before initiating the research, ethical clearance was granted by the Institutional Ethics Committee of Krishna Vishwa Vidyapeeth (Deemed to be University) (approval number: KVV/IEC/01(i)/2025; date: 29/01/2025). Furthermore, all study procedures were executed in strict adherence to the ethical guidelines established by the Declaration of Helsinki.

All enrollees provided voluntary written consent before participating, with full assurances of strict data confidentiality and the right to unconditionally withdraw from the investigation at any time without prejudice.

Assessment of KOA

Clinical diagnosis of bilateral KOA was established based on patient history and clinical examination, and the severity of medial compartment KOA was confirmed via standard weight-bearing anteroposterior radiographs of both knees. Radiographs were graded by an independent, blinded musculoskeletal radiologist according to the Kellgren-Lawrence classification system. Functional limitations and pain severity were assessed through standardized outcome measures. To assess bilateral involvement, data from both knee joints were collected; however, to avoid unit-of-analysis error, the primary analysis used the mean score across both limbs for each participant. In cases where a significant clinical discrepancy existed between limbs, the more symptomatic limb (defined by a higher baseline Visual Analog Scale (VAS) score) was utilized as the index knee for all functional outcome measures.

Outcome measures

Visual Analog Scale (VAS)

To determine the severity of symptoms, pain intensity was quantified via VAS (intraclass correlation coefficient (ICC)≈0.76-0.97) for measuring subjective pain perception, where "0" indicates no pain and "10" indicates intolerable pain. VAS is a subjective measure of the intensity of the participant's pain [17].

Timed Up and Go (TUG) Test

A widely accepted and reliable assessment (ICC=0.98), the TUG test, was implemented to evaluate functional mobility [17]. Participants followed a standardized protocol: rising from a chair, walking three meters, turning, and returning to a seated posture. The total time taken to complete the task was recorded using a stopwatch. Lower completion times indicated better functional mobility [18].

Manual Muscle Testing (MMT)

Muscle performance was objectively evaluated using MMT graded according to the standard Medical Research Council (MRC) scale, ranging from 0 (no visible or palpable contraction) to 5 (normal strength against maximum resistance) [19]. Testing focused on three key musculotendinous groups critical for lower extremity stability and gait: the quadriceps femoris, hamstrings, and hip abductors. To ensure reliability, the "make test" protocol was utilized, wherein the examiner provided progressive isometric resistance against the participant's maximal effort in standardized testing positions.

Treatment protocol

To maintain the internal validity of the eight-week intervention, participants were required to maintain stable baseline medication levels. Participants in both groups underwent a supervised rehabilitation protocol of thrice-weekly physical therapy interventions spanning eight consecutive weeks. Each session lasted approximately 45-60 minutes. The initiation of new analgesics, non-steroidal anti-inflammatory drugs (NSAIDs), or intra-articular injections was prohibited during the study. Daily physical activity and home exercise were monitored through weekly self-report diaries, and use of walking aids remained consistent with baseline status.

Group A (Conventional Physiotherapy)

Preparation (15 minutes): Therapeutic ultrasound (1 MHz, 1.5 W/cm^2^) was applied to the medial joint line for seven minutes, followed by moist hot packs for 10 minutes.

Stretching (10 minutes): Participants performed passive stretching of the gastrocnemius, hamstrings, and iliotibial (IT) band, consisting of three repetitions of a 30-second hold followed by a 15-second rest period.

Strengthening (25 minutes): This included quadriceps isometrics, straight leg raises (SLR), and weighted hip abductions (three sets of 10-12 repetitions).

Progression: Resistance was increased in 0.5 kg increments using ankle weights once participants could complete three sets of 12 repetitions.

*Group B (Experimental Group*)

This integrated approach combined central stabilization with biomechanical unloading.

Part 1 is the DNS protocol (25 minutes) which comprised three phases, namely, Phase 1 (weeks 1-2), isolated diaphragmatic breathing and IAP regulation in the three-month supine position (90/90); Phase 2 (weeks 3-5), transitioning to "baby rocking", "rolling", and "prone on elbows" to enhance joint centration; and Phase 3 (weeks 6-8), advanced postures including oblique sitting, tripod, and high kneeling. For intensity and progression, exercises were performed in three sets, with postures held for 5-10 respiratory cycles. Advancement to more complex postures occurred only when the therapist confirmed the maintenance of a neutral spine and stable IAP without accessory muscle compensation.

**Figure 2:**
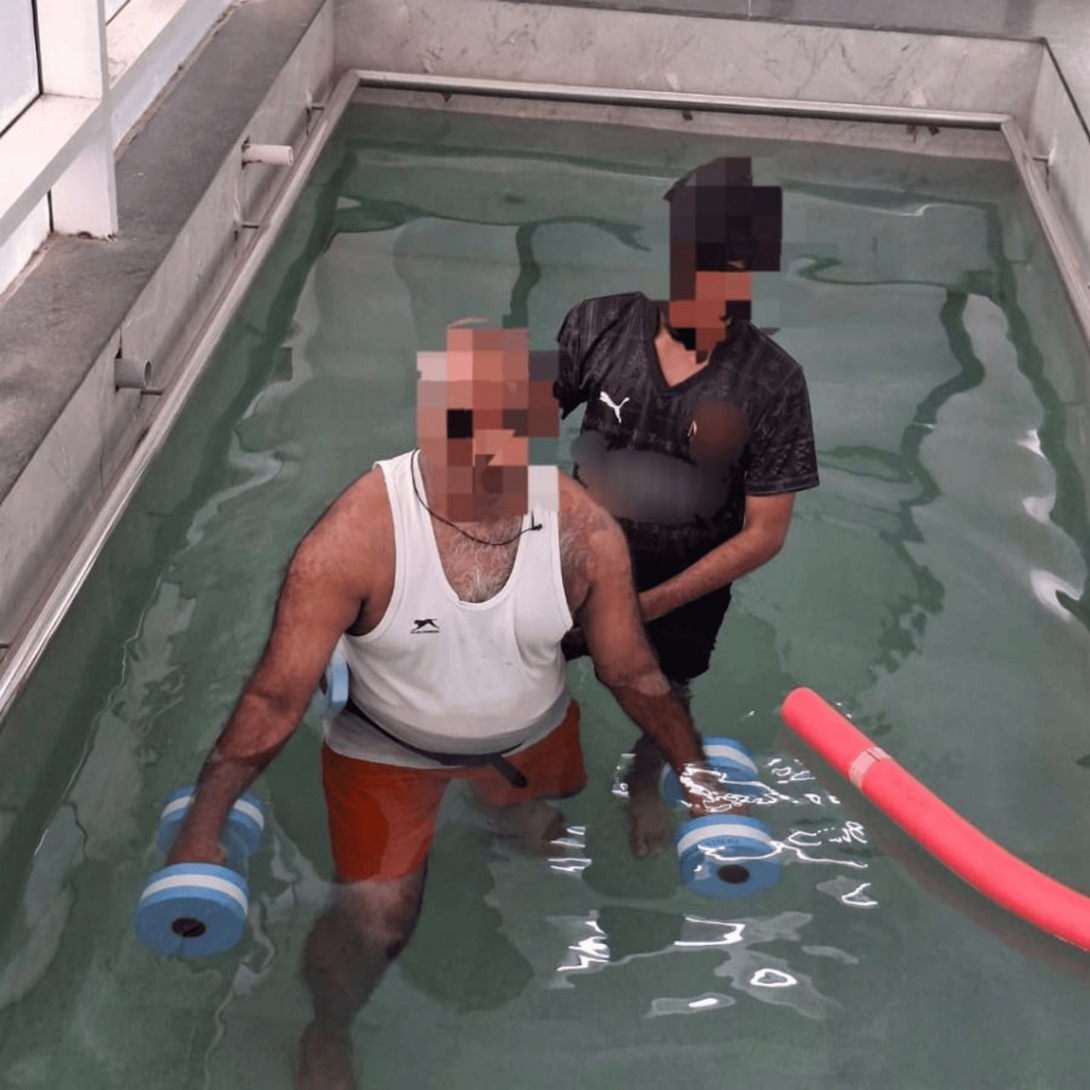
Aquatic dumbbell walking

**Figure 3:**
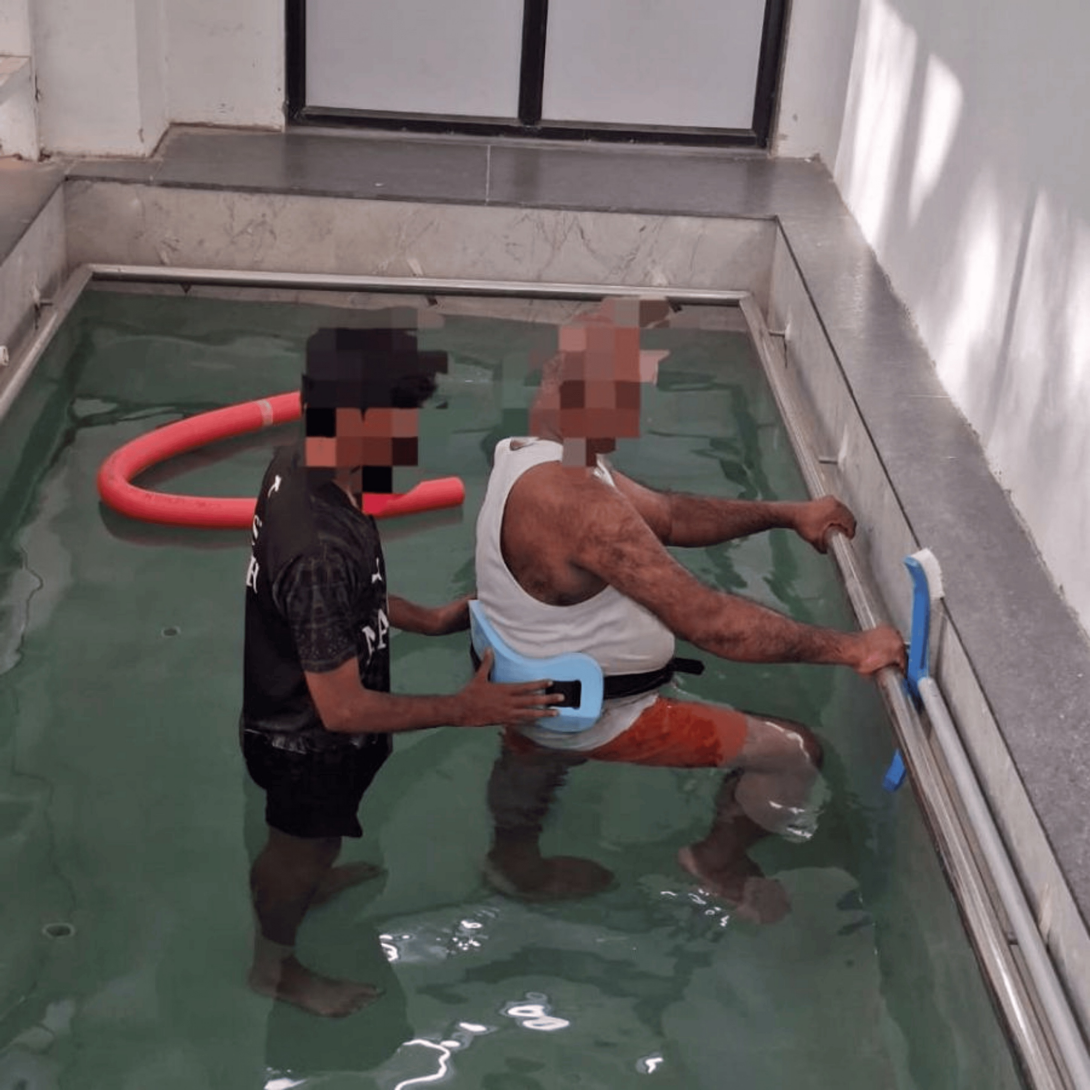
Aquatic supported knee flexion

Part 2 is the aquatic therapy protocol (25 minutes). The environment was a hydrotherapy pool maintained at 26-36°C, with the water depth at the hip/anterior superior iliac spine level to reduce weight-bearing. All exercises were performed under the direct supervision of a specialized aquatic exercise therapist (Figures 2-3). The exercises were kickboard and belt walking (forward/lateral), aquatic dumbbell multidirectional walking, semi-squats, and resisted knee flexion/extension. For intensity and progression, exercises were performed in three sets of 15 repetitions, with increased speed of movement (utilizing water viscosity) or the addition of larger aquatic fins/dumbbells to increase drag resistance as pain (VAS) decreased.

Statistical analysis

Quantitative analysis of the collected data was executed using IBM SPSS Statistics for Windows, Version 26.0 (IBM Corp., Armonk, New York, United States), in consultation with a professional statistician. To determine the therapeutic efficacy of the intervention within each group, a paired t-test was employed to identify significant changes in research parameters from baseline to post-treatment. Additionally, an unpaired t-test was utilized to compare the two groups, assessing for baseline differences and post-protocol variations. Data trends and variability were quantified by calculating the mean and standard deviation for all measured variables. In addition to evaluating statistical significance, the clinical relevance of the intervention was estimated by comparing the mean within-group and between-group changes against established minimal clinically important difference (MCID) thresholds for the VAS (1.5-2 points), TUG test (0.8-1.4 seconds), and MMT (approximately one full grade) [20-22]. Because a full responder analysis of individual trajectories was not feasible within the scope of this dataset, clinical relevance is interpreted strictly based on group-level averages.

## Results

Sixty participants fulfilled the study requirements and were included in the final data evaluation, with 30 participants in each group. No dropouts were reported during the intervention period. The study maintained a 100% completion rate with high adherence; importantly, no adverse events, such as increased joint effusion, significant pain exacerbation, or cardiovascular instability, were reported during either the land-based or aquatic interventions, confirming the safety profile of the protocols. The mean session attendance was 96% (range: 92-100%). No participants missed more than two sessions. No major protocol deviations or adverse events related to either the aquatic or land-based interventions were reported during the eight-week period.

Table 1 presents the baseline characteristics of the study participants, confirming that both groups were well-balanced prior to the intervention. Most participants were aged 49-56 years, with a slightly higher proportion of females in both cohorts. A vast majority were classified as overweight (BMI >25 kg/m²) and reported a symptom duration exceeding six weeks. The severity of osteoarthritis, indicated by Kellgren-Lawrence grades I and II, was evenly distributed. Statistical analysis revealed no significant between-group differences for age (p=0.95), gender (p=0.75), Kellgren-Lawrence grade (p=0.52), or BMI (p=0.60). Furthermore, symptom chronicity was comparable, with 80% of Group A and 76.7% of Group B reporting symptoms for over six weeks, ensuring both groups started from a statistically similar baseline.

**Table 1:** Demographic and clinical characteristics of the study participants Data is presented as n (%). Statistical significance between groups was determined using a chi-squared test. A p-value of >0.05 indicates no statistically significant difference between the groups at baseline. Group A: conventional physiotherapy (n=30); Group B: DNS and aquatic therapy (n=30) DNS: Dynamic Neuromuscular Stabilization; BMI: body mass index

Variable	Group A	Group B
Age (years)		
40-48	10 (33.3%)	9 (30%)
49-56	12 (40%)	13 (43.3%)
57-65	8 (26.7%)	8 (26.7%)
Gender		
Male	14 (46.7%)	13 (43.3%)
Female	16 (53.3%)	17 (56.7%)
Kellgren-Lawrence grade		
Grade I	12 (40%)	14 (46.7%)
Grade II	18 (60%)	16 (53.3%)
BMI		
Normal weight (<25 kg/m²)	7 (23.3%)	5 (16.7%)
Overweight (>25 kg/m^2^)	23 (76.7%)	25 (83.3%)
Duration of symptoms		
4-6 weeks	6 (20%)	7 (23.3%)
>6 weeks	24 (80%)	23 (76.7%)

Analysis of pre- and post-intervention data that showed both groups achieved statistically significant gains measured in all parameters is presented in Table 2.

**Table 2:** Within-group comparison of outcome measures Data is presented as mean±SD. Statistical significance within groups from baseline to post-test was determined using a paired t-test. Group A: conventional physiotherapy (n=30); Group B: DNS and aquatic therapy (n=30) SD: standard deviation; VAS: Visual Analog Scale; TUG: Timed Up and Go; MMT: Manual Muscle Testing; DNS: Dynamic Neuromuscular Stabilization

Outcome measure	Groups	Pre-test (mean±SD)	Post-test (mean±SD)	P-value	t-value
VAS	Group A	7.1±1.2	4.8±1.0	<0.01	3.12
	Group B	7.3±1.1	2.9±0.9	<0.001	12.45
TUG (seconds)	Group A	14.2±2.5	11.8±2.1	<0.01	3.34
	Group B	14.5±2.6	9.6±1.8	<0.001	8.76
MMT: quadriceps	Group A	3.2±0.4	3.8±0.5	<0.01	3.05
	Group B	3.1±0.5	4.4±0.4	<0.001	6.82
MMT: hamstrings	Group A	3.4±0.5	3.9±0.4	<0.01	2.98
	Group B	3.3±0.4	4.6±0.5	<0.001	11.20
MMT: hip abductors	Group A	3.1±0.4	3.6±0.5	<0.01	4.25
	Group B	3.2±0.5	4.4±0.4	<0.001	10.15

Pain (VAS)

In Group A, the mean VAS score decreased from 7.1±1.2 to 4.8±1.0 (p<0.01), yielding a mean reduction of 2.3 points. In Group B, pain levels showed a greater decline, with VAS scores dropping from 7.3±1.1 to 2.9±0.9 (p<0.001), yielding a mean reduction of 4.4 points.

Both groups achieved mean score reductions that exceeded the established MCID for VAS in KOA (typically 1.5-2 points), confirming clinically meaningful pain relief in both cohorts.

Functional mobility (TUG test)

Group A achieved a statistically significant reduction in TUG scores, decreasing from 14.2±2.5 seconds to 11.8±2.1 seconds (p<0.01), yielding a mean change of 2.4 seconds. Group B demonstrated a more pronounced average improvement, with TUG scores decreasing from 14.5±2.6 seconds to 9.6±1.8 seconds (p<0.001), reflecting a mean change of 4.9 seconds.

These group-level mean reductions in both cohorts surpass the estimated MCID for the TUG test in osteoarthritic populations (0.8-1.4 seconds), suggesting notable functional recovery on average for the participants.

Muscle performance (MMT)

Group A quadriceps strength improved from 3.2±0.4 to 3.8±0.5 (p<0.01), hamstrings from 3.4±0.5 to 3.9±0.4 (p<0.01), and hip abductors from 3.1±0.4 to 3.6±0.5 (p<0.01). Group B exhibited highly significant and notable gains: quadriceps strength increased from 3.1±0.5 to 4.4±0.4 (p<0.001), hamstrings from 3.3±0.4 to 4.6±0.5 (p<0.001), and hip abductors from 3.2±0.5 to 4.4±0.4 (p<0.001).

While specific MCID values for ordinal MMT grades are less rigidly standardized, the near one-grade or greater mean improvements observed, particularly in Group B, are generally accepted as clinically important for dynamic joint stability.

Table 3 presents the comparative results of the inter-group analysis, which revealed statistically significant variances in post-treatment outcomes.

**Table 3:** Between-group comparison of post-test outcomes Data is presented as mean±SD. Statistical significance for post-intervention outcomes between Group A and Group B was determined using an unpaired t-test. Group A: conventional physiotherapy (n=30); Group B: DNS and aquatic therapy (n=30) SD: standard deviation; VAS: Visual Analog Scale; TUG: Timed Up and Go; MMT: Manual Muscle Testing; DNS: Dynamic Neuromuscular Stabilization

Outcome measure	Group A (mean±SD)	Group B (mean±SD)	Mean difference	P-value	t-value
VAS	4.8±1.0	2.9±0.9	1.9	<0.001	7.74
TUG (seconds)	11.8±2.1	9.6±1.8	2.2	<0.001	4.36
MMT: quadriceps	3.8±0.5	4.4±0.4	0.6	<0.001	5.12
MMT: hamstrings	3.9±0.4	4.6±0.5	0.7	<0.001	5.99
MMT: hip abductors	3.6±0.5	4.4±0.4	0.8	<0.001	6.83

Pain (VAS)

The post-test VAS score in Group B (2.9±0.9) was markedly lower than the mean recorded in Group A (4.8±1.0). The resulting mean difference of 1.9 points between the groups was statistically significant (p<0.001) and approaches the upper limit of the MCID threshold itself, highlighting the potential clinical benefit of the combined intervention.

Functional mobility (TUG test)

The experimental group demonstrated better functional mobility. The mean difference of 2.2 seconds was statistically significant (p<0.001) and comfortably exceeded the TUG MCID threshold, indicating a favorable trend for the integrated protocol over conventional therapy on average.

Muscle performance (MMT)

Post-test comparisons demonstrated that Group B achieved significantly higher MMT scores than Group A in the quadriceps, hamstrings, and hip abductors (mean differences of 0.6-0.8 points; p<0.001). While the mean difference between groups is less than one full grade, it highlights that the experimental protocol consistently pushed participants toward the next functional strength category.

## Discussion

The primary objective of this investigation was to determine if the integration of DNS with aquatic therapy yields enhanced therapeutic results relative to standard physiotherapy for patients diagnosed with bilateral medial compartment KOA. Following the eight-week protocol, data from 60 subjects indicated that the experimental group outperformed the control group, demonstrating significantly more robust improvements in pain intensity and functional capacity. Highly significant statistical variances were specifically noted in VAS (p<0.001), TUG (p<0.001), and MMT grades (p<0.001).

Impact on pain (VAS)

One of the primary findings of the current research marked a reduction in pain levels in the experimental group (p<0.001). Specifically, Group B's mean VAS score dropped significantly from 7.3±1.1 to 2.9±0.9 (p<0.001), compared to Group A's decrease from 7.1±1.2 to 4.8±1.0 (p<0.01). This yielded a statistically significant outcome in the mean difference of 1.9 between the groups (p<0.001). Notably, the mean reductions in both groups exceeded the established MCID for VAS (1.5-2 points), suggesting that the changes are clinically meaningful for the average patient in these cohorts. Aquatic therapy contributes significantly to this pain reduction through the biomechanical advantage of buoyancy, which reduces compressive forces and varus torques that disproportionately load the medial compartment of the knee joint and allows for functional movements with reduced nociceptive stimulation. Such physiological processes align closely with previous literature; for instance, Coons et al. and Garbi et al. demonstrated the efficacy of aquatic walking and aquatic physiotherapy in reducing pain in KOA populations [23,24]. Simultaneously, DNS restores neuromuscular control and corrects dysfunctional movement patterns, leading to efficient load distribution, a benefit supported by Nurhayati et al., who highlighted the effectiveness of DNS in KOA [25].

Functional mobility (TUG test)

The TUG test assessed functional mobility and revealed that, although both cohorts exhibited post-intervention improvements, the experimental group demonstrated more distinct improvements in dynamic mobility and balance (p<0.001). The resulting mean difference of 2.2 seconds between the groups (p<0.001) exceeds the MCID for the TUG test (0.8-1.4 seconds), suggesting a meaningful clinical advantage of the combined intervention at the group level. Baliunas et al. emphasized that elevated mechanical loading on the knee joint during gait serves as a primary driver of functional decline in osteoarthritic populations [26]. The combined intervention combats this directly: aquatic therapy facilitates movement by reducing gravitational stress in a low-impact environment, while DNS enables smoother execution of functional tasks by reinforcing optimal movement patterns and postural stability which helps to correct the compensatory lateral trunk lean and altered gait mechanics often seen in bilateral medial compartment KOA.

Impact on muscle performance (MMT)

In addition, the shift in MMT grades within the experimental group reached a high level of statistical significance (p<0.001). For instance, Kang et al. [13] demonstrated that DNS training significantly enhances lower extremity strength profiles and functional stability by optimizing central motor control pathways. Furthermore, the magnitude of strength improvement observed in our experimental group aligns closely with the outcomes reported by Krishnan et al., who concluded that the accommodating resistance of aquatic environments allows for enhanced muscular endurance and strength adaptations in KOA populations compared to standard land-based protocols.

From a mechanistic perspective, the conjunction of DNS and aquatic therapy provides a dual approach targeting both peripheral biomechanical factors and central neuromuscular control. While aquatic therapy primarily reduces joint loading and facilitates movement, DNS enhances motor control and movement efficiency [27,28]. The statistically significant differences observed across all outcome measures (VAS: p<0.001; TUG: p<0.001; MMT: p<0.001) reinforce the functional impact of this combined approach.

It is essential to acknowledge that conventional physiotherapy also resulted in statistically significant improvements within its group (p<0.01), confirming its established role in managing bilateral KOA symptoms as seen in previous studies by Alkhawajah and Alshami [29]. However, the comparatively smaller magnitude of improvement suggests that conventional approaches alone may not sufficiently address neuromuscular deficits and joint loading factors to the same extent as a combined intervention.

Strengths, limitations, and future scope

A primary methodological strength of this investigation is the synergistic application of DNS and aquatic therapy, creating a comprehensive rehabilitative paradigm that targets both central motor pathways and peripheral joint biomechanics. The aquatic environment provided a mechanically advantageous, low-gravity setting to mitigate compressive tibiofemoral forces, thereby facilitating the safe restoration of physiological movement patterns dictated by DNS principles. The substantial statistical significance observed across all outcome metrics, including the VAS, TUG test, and MMT, underscores the high clinical efficacy of this multimodal intervention for managing bilateral medial compartment KOA.

Despite these promising findings, certain methodological constraints warrant consideration regarding the external validity of the results. A primary limitation of this study was the inability to implement participant or assessor blinding, which is often unavoidable in rehabilitation trials comparing distinct aquatic and land-based environments. While steps were taken to ensure participants remained unaware of the study's specific hypotheses and the alternative group's protocol, the physical nature of the interventions precluded true blinding to the treatment setting. Furthermore, as an integrated protocol, this study design cannot isolate the individual therapeutic contributions of DNS versus aquatic therapy. The trial was executed at a single clinical center with a finalized cohort of 60 participants. Importantly, while the group-level mean changes exceeded MCID thresholds, the lack of a full responder analysis or individual trajectory mapping means that we cannot account for inter-individual variability. Consequently, these findings should be interpreted as preliminary and suggestive rather than definitive guarantees of individual patient outcomes.

The intervention protocol was confined to an eight-week duration without subsequent longitudinal tracking, precluding definitive conclusions regarding the long-term survivability of the functional gains. Furthermore, the reliance on patient-reported outcomes and clinician-graded scales, including the inherent limitation of treating ordinal MMT grades as continuous data for statistical analysis, constrains the precise biomechanical interpretation of the observed clinical improvements. While this approach effectively illustrates group-level progress, the absence of objective biomechanical quantification, such as surface electromyography, 3D kinematic analysis, or advanced objective dynamometry, underscores a limitation in capturing underlying physiological mechanisms. Future studies should consider reporting the full distribution of ordinal grades or utilizing these objective technologies to further validate and refine the clinical findings reported here.

To substantiate these findings, future research imperatives should prioritize large-scale, multi-center randomized controlled trials and pre-registered protocols incorporating extended longitudinal follow-ups to evaluate the sustainability of therapeutic outcomes over time. The integration of advanced, objective biomechanical instrumentation will be critical for elucidating the precise neuromuscular and kinematic mechanisms underlying the combined DNS and aquatic therapy protocol. Additionally, stratifying subsequent study cohorts by varying degrees of radiographic joint degeneration will be essential to delineate the clinical utility of this intervention across the full pathological spectrum of KOA, ultimately informing standardized, evidence-based conservative management guidelines.

## Conclusions

This study provides preliminary evidence that the conjunction of DNS and aquatic protocols may offer enhanced clinical outcomes compared to routine physiotherapy for patients with bilateral medial compartment KOA. By evaluating the mean changes against established MCID thresholds, the combined intervention demonstrated clinical relevance at the group level, specifically in alleviating pain and enhancing functional mobility. However, given potential inter-individual variability, these results should be viewed as suggestive trends.

Therefore, a multimodal rehabilitation approach combining these therapies should be strongly considered in clinical practice for the optimal management of KOA. To build upon these findings, future research should incorporate long-term follow-ups, larger sample sizes, and objective biomechanical assessments to further validate the sustained effectiveness and underlying mechanisms of combined DNS and aquatic therapy interventions.
